# Rethinking natural hazards research and engagement to include co-creation with Indigenous communities

**DOI:** 10.1038/s44304-024-00034-7

**Published:** 2024-11-22

**Authors:** Thomas J. Jones, Harry Nyce, Yannick Le Moigne, Glyn Williams-Jones, Deanna Nyce

**Affiliations:** 1 Lancaster Environment Centre, Lancaster University, Lancaster, UK; 2 Wilp Wilx̱o’oskwhl Nisg̱a’a Institute, Gitwinksihlkw, BC Canada; 3 Natural Resources Canada, Geological Survey of Canada, Vancouver, BC Canada; 4 Centre for Natural Hazards Research, Department of Earth Sciences, Simon Fraser University, Burnaby, BC Canada

**Keywords:** Volcanology, Natural hazards, Geography

## Abstract

Indigenous peoples are widely affected by natural hazards and their history and knowledge can directly inform on past events and mitigation strategies. Here we show how effective co-creation of resources and bi-lateral knowledge exchange between natural hazard researchers and local Indigenous communities provides an effective, equitable, and sustainable way to conduct research.

## Introduction

**Fig. 1:**
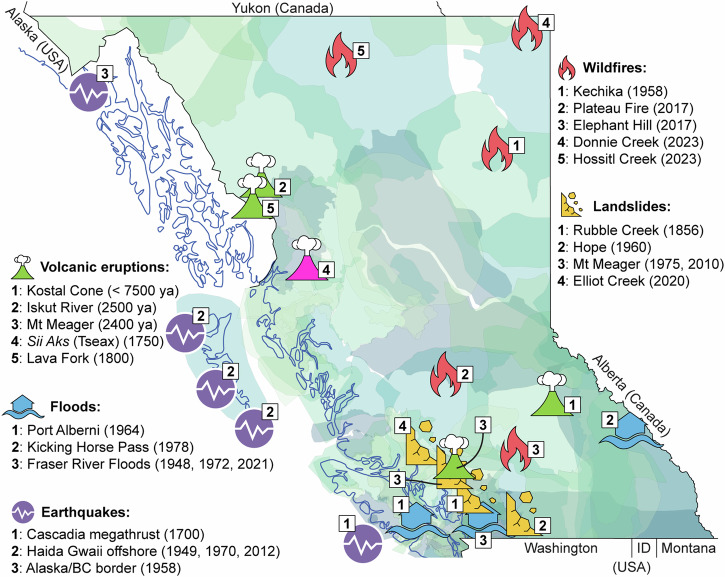
Natural hazards impacting Indigenous peoples in British Columbia, Canada. The map view encompasses the entire Canadian province of British Columbia, bordered on the East by Alberta, the South by Washington, Idaho (ID) and Montana, the Northeast by Alaska and the North by the Yukon. Symbols represent recent large volcanic eruptions, earthquakes, landslides, wildfires and floods in British Columbia^65^. The magenta-coloured volcano symbol marks the location of the *Sii Aks* (also referred to with the past spelling of Tseax) volcanic eruption in the mid-1700s, and a focal topic of this article. Indigenous Territory outlines^66^ (shades of blues and greens) are approximate and do not represent official boundaries of any communities. This figure was created by the authors using ArcGIS Pro.

Internationally there is no single agreed definition of Indigenous peoples, and here we use Indigenous as an all-encompassing international term (in Canada this includes First Nations, Métis and Inuit). The United Nations Office for Disaster Risk Reduction (UNDRR) reports^1^ that 476 million people in more than 90 countries identify as Indigenous and ~20% of the Earth is covered by Indigenous territories. Consequently, Indigenous peoples globally live at risk from natural hazards (e.g., volcanic eruptions, landslides, earthquakes) and also receive benefits from living in active geological areas (e.g., fertile soils, tourism, geothermal power). According to 2021 Canadian census data^2^, in the Province of British Columbia (B.C.) there are 290,210 people who identify as Indigenous, with 180,085, 97,865 and 1725 people self-identifying as First Nations, Métis and Inuit, respectively. All these people are susceptible to natural hazards and, as illustrated by Fig. 1, the largest volcanic eruptions, earthquakes, landslides, wildfires, and floods in British Columbia, Canada have all affected Indigenous territories. Thus, given the global spatial overlap between Indigenous peoples and natural hazards, and the need for meaningful collaboration, both research and Indigenous local knowledge should be shared for mutual benefit. Here, we outline how the Nisg̱a’a First Nation and volcanology researchers have initiated and maintained a fruitful collaboration with bi-lateral knowledge exchange and resource co-creation. Furthermore, this comment article is co-written by non-Indigenous volcanology researchers based at universities (Jones and Williams-Jones) and a government organisation (Le Moigne) and by Indigenous scholars of the Nisg̱a’a First Nation, based at a post-secondary education establishment (Nyce and Nyce Jr.).

## Outcomes and values of co-creation

It is common for research scientists to conduct field-based studies on natural hazards in geographical areas where they are not a resident, and globally there is a growing awareness of the need for, and mutual benefits of, meaningful collaboration with local scientists and agencies^3^. However, relatively little recognition has been given to such collaboration as applied to Indigenous communities. There is little clarity around how an appropriate relationship is initiated and established, and the ways in which visiting researchers share knowledge and perform outreach with local Indigenous communities (e.g., the local communities, industries, schools). Here we discuss the benefits and values of bi-lateral exchange and resource co-creation versus simple unidirectional outreach (e.g., public lecture, providing an information poster) and share some insights based on the experiences shared by the Indigenous and non-Indigenous authors of this comment.

There are many different resources that can be co-created between researchers and Indigenous communities. For the purposes of disseminating knowledge and history about natural hazards and past events, resource examples include information boards, posters, school activities, museum displays, online media, artwork, and audio tours. Irrespective of the resource type, co-creation and development has multiple benefits. The knowledge exchange is bi-lateral, and in many cases, Indigenous Knowledge directly informs on risk mitigation and hazard perception^4^. The co-creation process also allows for reciprocation—the research project directly benefits from broader perspectives and opportunities are developed for the local community^5^. Lastly, the co-created resources build capacity in a sustainable way. If the end users of a resource are involved in its design, development, and creation, it is more likely to be used effectively and shared amongst the community.

## The Nisg̱a’a First Nation and volcanology researchers

In the mid-1700s CE, *Sii Aks* (Tseax) volcano erupted and was responsible for the deaths of up to 2,000 people of the Nisg̱a’a Nation, ranking it as Canada’s second-worst natural disaster^6,7^. *Sii Aks* is Nisg̱a’a for new (*Sii*) water (*Aks*) and in Nisg̱a’a history, the eruption caused the emergence of a new river. More specifically, the location of the eruption is known as *Wil Ksi Baxhl Mihl* which means ‘where the fire ran out’ and there is direct reference to the *Sii Aks* eruption in Nisg̱a’a history. Years later, a relatively small number of research articles were published about the eruption and the geology^8–12^, which, to our knowledge, involved little meaningful engagement or consultation with the Nisg̱a’a Nation.

The Nisg̱a’a Final Agreement came into effect in May 2000 and represented the first modern treaty in British Columbia to incorporate inherent rights to self-government^13^. One result of discussions leading to the Treaty was the establishment of *Anhluut’ukwsim Lax̱mihl Angwinga’asanskwhl Nis*g̱*a’a* (Nisga’a Memorial Lava Bed Park) on April 29, 1992. It is the first British Columbia Provincial Park to be co-managed with a First Nation. This is particularly relevant here as *Sii Aks* volcano and its erupted products are located within this park.

**Fig. 2:**
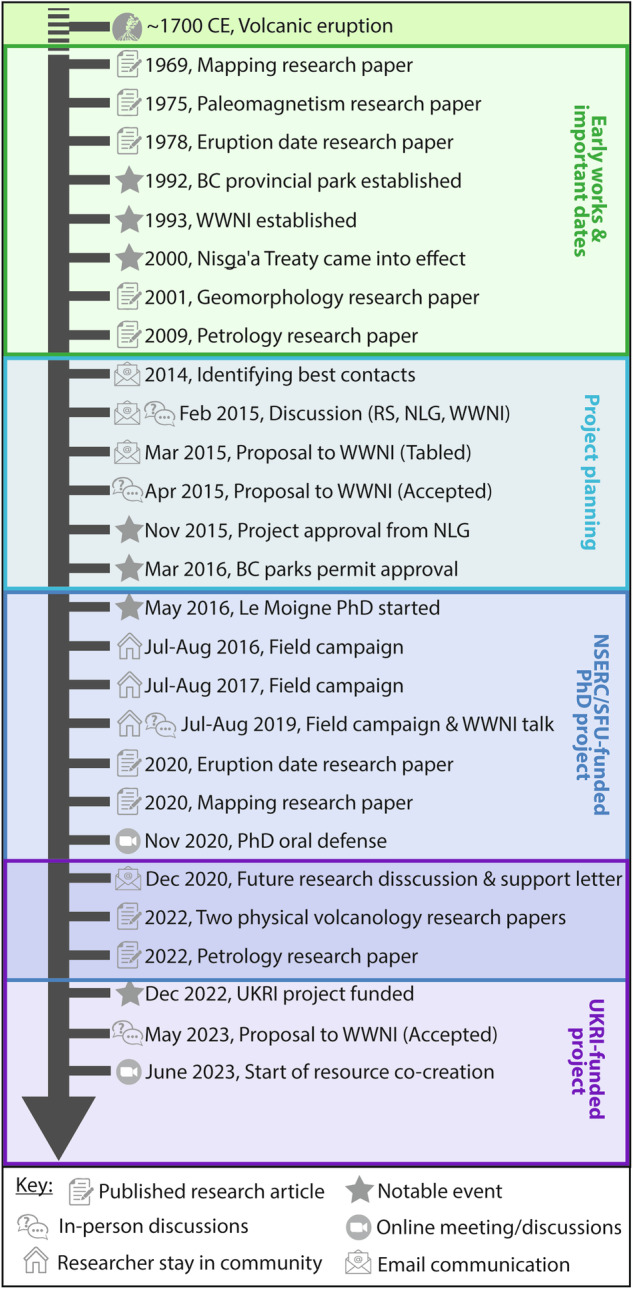
Overview timeline of volcanology research scientist engagement with the Nisag̱’a Nation. BC British Columbia, RS Research scientist, NLG Nisg̱a’a Lisims Government, WWNI *Wilp Wilx̱o’oskwhl Nis*g̱*a’a* Institute, NSERC Natural Sciences and Engineering Research Council of Canada, SFU Simon Fraser University, UKRI United Kingdom Research and Innovation.

Supported by the timeline in Fig. 2, we briefly document the exchange and evolving relationship between members of the Nisg̱a’a Nation and volcanology research scientists (i.e., both groups being represented as authors on this article). This started in 2014 when research scientists interested in studying the *Sii Aks* eruption first carefully identified appropriate groups (or stakeholders) from the Nisg̱a’a Nation and then started both in-person and electronic discussions with representative members of the Nation’s government, Nisg̱a’a Lisims Government (NLG) and the *Wilp Wilxo’oskwhl Nisga’a* Institute (Nisga’a House of Wisdom; WWNI). The WWNI serves as host of the Nisg̱a’a Research Protocol as well as the Nisg̱a’a centre for research and post-secondary education. These initial and early conversations ensured that all goals and priorities were of mutual benefit. The research proposal was then written by the research scientists and submitted to the WWNI electronically in March 2015. The proposal was initially tabled due to concerns about reference to oral history and stories, specifically concerns were raised about science trying to prove or disprove oral stories and issues surrounding the sharing of stories by researchers or groups to whom the stories do not belong. Although this was never intended, this was explicitly made clear, expressing that Indigenous Knowledge and science are both valuable data and represent information through different lenses^14,15^. The proposal was then resubmitted in-person with an accompanying presentation and discussion the following month and approved by the WWNI (Fig. 2). The project, started in 2016, aimed at understanding the physical characteristics of the eruption (e.g., volume, age, duration); and it was funded by the Natural Sciences and Engineering Research Council of Canada (NSERC) and Simon Fraser University (SFU). Key community engagements included three field seasons where the PhD researcher (Le Moigne) and field assistants stayed with community members for ~2 months each time, an information sharing presentation at the WWNI, and a public online seminar. Informed jointly by *Nis*g̱*a’a Adaawaḵ* (Nisg̱a’a stories and histories) and the physical field evidence, this project supported co-creation of an eruption chronology, a map, and a short documentary film. In all cases, following discussions surrounding contributions, members of the Nisg̱a’a Nation were either directly acknowledged in written text, credited by institutional affiliation (e.g., WWNI logo), or in the case of the documentary, community members appeared directly as named individuals.

Building on the fundamental research conducted^6,16–18^ at *Sii Aks* and the WWNI-Researcher relationships, in 2020, a second research project involving community outreach, further bilateral knowledge exchange and resource co-creation was discussed and jointly submitted to UK Research and Innovation (UKRI) for funding consideration. This was funded and started in 2022. The research team then visited the WWNI in May 2023 to present and discuss the proposed research, this was approved, and resource co-creation began the following month (Fig. 2). To date, and on direct feedback from WWNI and NLG, we have co-created teaching materials and a new curriculum at the WWNI bringing together the volcanology and the cultural aspects of *Sii Aks*.

## Reciprocal benefits

**Fig. 3:**
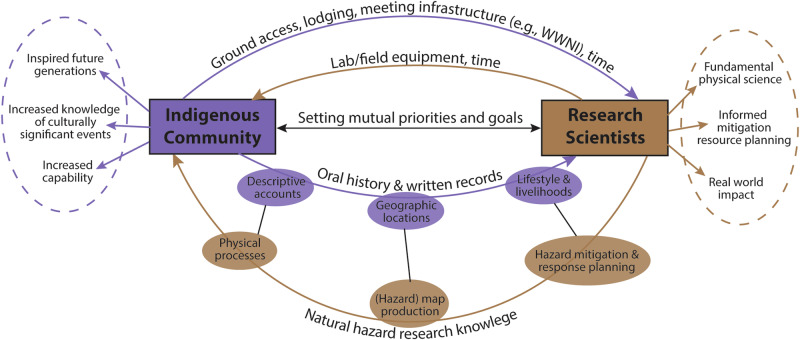
The benefits of bi-lateral exchange between Indigenous communities and research scientists in the context of natural hazards. The purple and brown arrows represent knowledge and resource exchange between Indigenous communities and research scientists, respectively. The dashed circles represent outcomes relevant to the broader community.

Based on our experience, we highlight several mutually beneficial exchanges involving both Indigenous communities and research scientists working on natural hazards. These reciprocal benefits are applicable globally and are not specific to the natural hazard or the individual researcher-Indigenous community relationship. Initial interaction and exchange ensure that all parties have aligned priorities, and the outcomes of any proposed activities are of mutual benefit (Fig. 3). Logistically, local Indigenous partners can facilitate ground access, accommodation, and infrastructure (such as meeting rooms). Research scientists on the other hand can often provide access to unique, highly specialist field and laboratory equipment. Only with this equipment can some investigations of a previous hazardous event be undertaken (e.g., isotopic dating, geobarometry). The bi-lateral exchange of knowledge in the form of oral stories, written records and research science can cover many aspects, for example, descriptive accounts from past natural hazard events can directly support and ground-truth physical processes (e.g., eye-witness accounts of eruption column heights or flood depths, the duration of events). Identifying the geographic locations of culturally important sites and settlements supports (hazard) map production that is attentive to the needs of the local community. Furthermore, understanding typical community lifestyles, livelihoods, and beliefs aids in understanding the impact of past events and informs on future natural hazard mitigation and effective response planning^19,20^. Bi-lateral exchange can therefore have direct and tangible outcomes for all involved (Fig. 3).

## Key lessons

- Before any research project starts, it is vitally important to scope out all parties, organisations, and community groups and provide them equal opportunity to contribute to the project design, planned objectives, goals, and priorities. In this way co-creation is embedded from the very outset.
- Navigating internal structures has been straightforward, in this case, thanks to the WWNI—an institution that serves as the Nisga’a research and post-secondary education centre. Meetings hosted by the WWNI have enabled external researchers to engage with Nisg̱a’a Elders, the NLG and broader community members. In cases where such a structure does not exist, it is important to identify a focal person or group that can facilitate engagement with appropriate parties.
- Measures and assurances need to be in place to ensure Indigenous data sovereignty and that Indigenous Knowledge is appropriately and respectfully acknowledged^21^. Whilst the exact framework adopted should be discussed with the Indigenous community and science researchers, several protocols and networks already exist. These include, the Ownership, Control, Access, Possession principles from Canada^22^, the United States Indigenous Data Sovereignty Network^23^, the *Te Mana Raraunga* Indigenous Data Sovereignty Network’s Charter^24^ from Aotearoa New Zealand. This is another reason that co-creation should occur at the very outset, in the project development stage, to ensure that Indigenous interests are asserted in relation to data^25^.
- Relationship building and project planning takes time and investment from all parties. In our case ~9 years from initial discussions to the start of resource co-creation.
- Face to face, in-person meetings are preferred and vital to initiate, establish and build relations during the project development and approval stage. Since the COVID-19 pandemic, the use of online conferencing is more frequent and makes regular meetings more achievable and environmentally friendly, however, these online exchanges must be supported by in-person visits.
- For the case of the *Sii Aks* (Tseax) eruption, very little volcanological science was known before our recent work. Thus, before we could contribute volcanology knowledge, we needed to perform a series of field studies. However, even in this ‘heavy research’ phase, bilateral knowledge exchange was possible and advantageous. Simultaneous meetings and residential stays with community members allowed researchers to hear *Nis*g̱*a’a Adaawaḵ* (Nisg̱a’a stories and histories) about the eruption that aligned with and extended our knowledge from field-based observations.
- Irrespective of the outcome of any co-created products/resources, the processes of co-designing a project plan and grant proposal are highly valuable and should not be understated. Rich bi-lateral knowledge exchange can occur during this time and set the foundation for long term reciprocal collaboration.

## Key challenges

Despite initiating and maintaining a partnership, some overarching challenges exist which remain to be resolved. Indigenous peoples have their own languages and thus co-created material should be produced in the local Indigenous language as a minimum. This, along with the time spent on co-creation activities, creates additional demands on the Indigenous community. As we have shown, it is important to develop and maintain relationships over long time periods with the same external researchers. For this to be possible, funders need to support long-term (i.e., multi-year, perhaps even decadal) engagement plans. This would also allow project members to take advantage of serendipitous opportunities (e.g., local festival, community event) and would support a natural evolution of timely project ideas that are directly informed by community needs and priorities.

## Recommendations and looking forward

Resource co-creation with local Indigenous communities is a highly valuable and effective means^5,26–28^ to conduct community outreach and engagement that is currently underused^29^. Individual Indigenous groups in Canada have Research Protocols to govern research activities. Globally there are established constructs to weave both Indigenous and non-Indigenous Knowledge systems, that can help draw on the strengths of both, whilst maintaining Indigenous data sovereignty. Examples include, but are not limited to, concepts such as *Etuaptmumk* or Two-Eyed Seeing^30^, *He awa whiria* (sometimes referred to as the braided river approach)^31–33^, and *Kūlana Noiʻi*^34,35^. In all cases, the blending or weaving of such knowledge systems may potentially yield a more powerful solution/outcome than a single knowledge system combined^32^. In any of the frameworks, even when knowledge convergence occurs, there is no attempt to homogenise or assimilate knowledge into a single system^36–38^. Further, it should be noted that challenges and tensions exist for scholars working between these two worlds^39^, forming the concept of an ‘edgewalker’—people who remain true to their spiritual or cultural traditions and also engage in the mainstream^40^.

In the discipline of volcanology, where volcanic eruptions constitute the natural hazard, there have been previous examples of knowledge generation and engagement using Indigenous Knowledge systems. This includes the use of oral traditions, language, and artwork as sources of information about past eruptions, interactions with the broader environment, and their hazard footprint^41–53^, the use of Indigenous Knowledge systems during eruption crises^54^, the co-creation of emergency plans^4^, the design and implementation of educational programmes^55–57^, the management and sustainability of geoparks^58,59^ and the use of local Indigenous Knowledge in disaster risk reduction^60–63^ (DRR), in line with the agenda within the 2015 Sendai Framework for DRR^64^. Despite these efforts, improvements can always be made, especially in performing genuine co-creation of knowledge.

For any research on natural hazards, we suggest that subject to any Research Protocol, with the appropriate time investment, relationship building and forward planning, research should be co-developed with the local Indigenous community from the very outset. This will ensure that both parties understand each other’s needs and priorities and can work together to achieve a mutually beneficial outcome with real, direct, and meaningful impact. This process of relationship building however, takes time (potentially years), and requires financial support. This includes travel for in-person discussions and funding for the time contributed by Indigenous participants (or some other agreed-upon, in-kind compensation). Thus, research funding councils should ensure that networking and exchange style grants are eligible for Indigenous community partners and not solely limited to traditional academic institutions.

## Data Availability

No datasets were generated or analysed during the current study.
